# The cancer glycocode as a family of diagnostic biomarkers, exemplified by tumor-associated gangliosides

**DOI:** 10.3389/fonc.2023.1261090

**Published:** 2023-10-26

**Authors:** Ali Nejatie, Samantha S. Yee, Anna Jeter, Horacio Uri Saragovi

**Affiliations:** ^1^ Center for Translational Research, Lady Davis Research Institute-Jewish General Hospital, Montreal, QC, Canada; ^2^ Pharmacology and Therapeutics, McGill University, Montreal, QC, Canada; ^3^ Department of Obstetrics and Gynecology, University of Chicago, Chicago, IL, United States; ^4^ AOA Dx, Boston, MA, United States; ^5^ Ophthalmology and Vision Science, McGill University, Montreal, QC, Canada

**Keywords:** biomarker, diagnosis, cancer, prognosis, early stage, screening, ganglioside

## Abstract

One unexploited family of cancer biomarkers comprise glycoproteins, carbohydrates, and glycolipids (the Tumor Glycocode).A class of glycolipid cancer biomarkers, the tumor-marker gangliosides (TMGs) are presented here as potential diagnostics for detecting cancer, especially at early stages, as the biological function of TMGs makes them etiological. We propose that a quantitative matrix of the Cancer Biomarker Glycocode and artificial intelligence-driven algorithms will expand the menu of validated cancer biomarkers as a step to resolve some of the challenges in cancer diagnosis, and yield a combination that can identify a specific cancer, in a tissue-agnostic manner especially at early stages, to enable early intervention. Diagnosis is critical to reducing cancer mortality but many cancers lack efficient and effective diagnostic tests, especially for early stage disease. Ideal diagnostic biomarkers are etiological, samples are preferably obtained via non-invasive methods (e.g. liquid biopsy of blood or urine), and are quantitated using assays that yield high diagnostic sensitivity and specificity for efficient diagnosis, prognosis, or predicting response to therapy. Validated biomarkers with these features are rare. While the advent of proteomics and genomics has led to the identification of a multitude of proteins and nucleic acid sequences as cancer biomarkers, relatively few have been approved for clinical use. The use of multiplex arrays and artificial intelligence-driven algorithms offer the option of combining data of known biomarkers; however, for most, the sensitivity and the specificity are below acceptable criteria, and clinical validation has proven difficult. One strategic solution to this problem is to expand the biomarker families beyond those currently exploited. One unexploited family of cancer biomarkers comprise glycoproteins, carbohydrates, and glycolipids (the Tumor Glycocode). Here, we focus on a family of glycolipid cancer biomarkers, the tumor-marker gangliosides (TMGs). We discuss the diagnostic potential of TMGs for detecting cancer, especially at early stages. We include prior studies from the literature to summarize findings for ganglioside quantification, expression, detection, and biological function and its role in various cancers. We highlight the examples of TMGs exhibiting ideal properties of cancer diagnostic biomarkers, and the application of GD2 and GD3 for diagnosis of early stage cancers with high sensitivity and specificity. We propose that a quantitative matrix of the Cancer Biomarker Glycocode and artificial intelligence-driven algorithms will expand the menu of validated cancer biomarkers as a step to resolve some of the challenges in cancer diagnosis, and yield a combination that can identify a specific cancer, in a tissue-agnostic manner especially at early stages, to enable early intervention.

## 1 Introduction

The tumor glycocode includes Tumor-Associated Carbohydrate Antigens (TACAs) that are carbohydrates that are displayed on cancer cell surfaces as glycans, glycoproteins and glycolipids, or can appear in soluble forms in circulation. Gangliosides are a subclass of glycolipids that contain one or more sialic acid residues. The specific TMGs, GD2 and GD3, are part of the tumor glycocode or “sugar code” in cancer (1–6). Gangliosides are the focus of this review, as they exemplify a new class of diagnostic biomarkers for cancer.

Historically, glycolipids in general have not been explored as diagnostic biomarkers because they are difficult to quantify using conventional proteomics or genomics techniques. Moreover, glycolipids have complex pathways for biosynthesis, metabolism, and catabolism. Methods that enable exploitation of these markers are now emerging. Thus, this review focuses on the growing evidence that TMGs represent a novel class of biomarkers with great potential in the early diagnosis of specific types of cancer (1–3).

Cancer is the second leading cause of death in the United States. The American Cancer Society estimates that over 1.9 million individuals will be diagnosed with cancer in 2023 and about 609,820 individuals will die of the disease (7). Efforts to reduce cancer mortality focus on improving cancer prevention, cancer screening (detecting cancer in asymptomatic individuals), early cancer diagnosis (detecting cancer in symptomatic individuals), and cancer treatment. Early cancer diagnosis enables rapid intervention when cancer is at an early stage and treatment outcomes are optimal (8). This has been shown to reduce mortality and morbidity, improve quality of live, and reduce health care costs and utilization (9). Conversely, delays in cancer diagnosis are associated with cancer progression from early stage to late stage, untreatable, disease (10). Thus, the lack of efficient early diagnosis for many types of cancer epitomizes an unmet and urgent medical need.

The identification and implementation of early diagnostic measures has improved outcomes for many cancers, including cancer of the colon (11), lung (12), breast (13), cervical (14) and prostate (15). Conversely, absence of early diagnosis worsens outcomes. Studies showed that a 3-month delay in cancer diagnosis due to the COVID-19 pandemic led to an overall 18% reduction in net survival (10), with breast (22%), lung (20%), ovarian and cervical (50%) and colorectal and stomach (67%) cancers showing the greatest negative impact. The Centers for Disease Control and Prevention supports screening for breast, cervical, colorectal and lung cancers.

However, in cancers which do not have accurate early diagnostic tests [e.g. ovarian and pancreatic (11–14, 16–18)], patient survival rates have remained persistently low with five-year survival of only 50% and 12.5% (9) in spite of introduction of new treatment regimens. Currently available methods for screening of ovarian, pancreatic, prostate, testicular, and thyroid have not reduced cancer deaths. Hypothetical modeling of improved screening and early detection would yield an anticipated potential reduction of five-year mortality of 26-39% (16).

## 2 Optimal features for cancer biomarkers

An optimal cancer biomarker should be etiological to disease mechanisms, stable, invariant, and with uniform expression in most/all cancer nodules within a patient, should not downregulate in cancer cells, or in the cancer cells that remain or that recur following therapy, and should be accessible or measurable in a patient using non-invasive standard procedures such as liquid biopsy (e.g., a blood sample). Unfortunately, cancer biomarkers that meet all these features are very rare.

### 2.1 Etiological versus surrogate biomarkers

Biomarkers are defined by the Food and Drug Administration (FDA) as characteristics of the body that you can measure that are a direct measure of how a patient feels, functions, or survives (19). A biomarker is diagnostic of a disease when it is detectable in disease but is low/absent or is not functionally relevant in a healthy cell or healthy individual.

Biomarkers can be surrogate or etiological to the disease. The presence of a surrogate marker may be associated with the presence of a disease, and this is an indirect form of measuring a disease that can lead to poor sensitivity and/or selectivity and unacceptable false positives or false negatives. Thus, surrogate biomarkers are less desirable for diagnostic purposes.

In contrast, etiological biomarkers have a function that is directly relevant or causative of disease or disease progression, and therefore afford a clinically meaningful endpoint. Examples of etiological markers include overexpressed or mutant ERBB2/HER2 (20) in breast cancer, mutant EGFR in lung cancer (21), and mutant *NRAS* or *BRAF* in melanoma (22). Etiological biomarkers are expected to be expressed in a stable manner, because a cancer cell cannot readily eliminate or downregulate it without suffering a disadvantage (e.g. loss of the function that the marker provides) although downregulation of an etiological marker may be compensated by mutations or alternative pathways. Moreover, etiological biomarkers are expected to be expressed uniformly within a patient, even though cancer cells are phenotypically and genetically heterogeneous. Paradoxically, however, etiological protein biomarkers such as ERBB2/HER2, mutant EGFR, mutant BRAF and many others are expressed in a heterogeneous manner and often do downregulate expression leading to drug resistance.

### 2.2 Tissue biopsy versus liquid biopsy

Targets that are exclusively cell-associated (intracellular or membrane-bound in cancer cells) are available for study only through tissue biopsy, which may be invasive and require specialized equipment and procedures. Tissue biopsies are restricted to small sample size, offer a window limited to the tissues collected, and yield limited information regarding genetic heterogeneity within the primary tumor or the metastasized secondary tumors.

However, many cellular targets may also be found in soluble form if they are shed or secreted, or because of a cancer-associated metabolic or physiological process, or after they are proteolytically cleaved, or if they are released by the cancer cell in the form of exosomes or extracellular vesicles (EVs). These soluble markers are available for study using a liquid biopsy such as in blood, urine, tears, saliva, or cerebrospinal (CSF) fluids. Liquid biopsy sample collection is less invasive and more convenient, can be repeated over time, and is independent of tumor location, hence the diagnostic power and coverage of this method is very high (23, 24). Liquid biopsies are emerging as the fastest growing diagnostic tool for cancer and inflammation. Examples include quantification of circulating tumor DNA (ctDNA) (25), RNA, cell-free DNA (cfDNA) (26), DNA methylation and fragmentation (27), and targeted proteomics as approaches for tissue-agnostic biomarkers. Emerging pan-cancer tests are reported to detect up to 3 or 4 tumor types (based on nucleic acids mutations or methylation states), but fall below a threshold diagnostic coverage of over 50% of the patients in the top 30 tumor types (26).

As the FDA issued the first tissue-agnostic (or biomarker-driven) cancer drug approval (28), we anticipate that tissue-agnostic etiological biomarkers will become more interesting clinically and commercially. In this review we put forth the concept that TMGs are biomarkers that meet all the desirable characteristics described above: they are etiological to disease mechanisms, stable, invariant, and with uniform expression in most/all cancer nodules within a patient, do not downregulate following therapy, and are accessible in a liquid biopsy. Despite these favorable characteristics, TMGs remain under-exploited for diagnostics.

### 2.3 Traditional classes of cancer biomarkers. Proteins and nucleic acids

Cancer biomarkers have traditionally focused on measurements of abnormal proteins that are expressed *de novo*, or proteins that are over-expressed, mutated, or dysregulated; and on measurements of abnormal nucleic acids (DNA, mutant DNA, mRNA, miRNA), their methylation state, or nuclear structure. Approaches for discovery of novel targets or markers include proteomics using Mass Spectrometry (29) and genomics (30) through Next Generation Sequencing (NGS) and PCR (31), whole genome sequencing, or sequencing mRNA transcripts (32), and metabolomics (33). These “omics” techniques can be applied to tissue biopsies and to liquid biopsies, and have advanced the field by discovering many new biomarkers. The vast array of data obtained is refined qualitatively by incorporating artificial intelligence and bioinformatic platforms to the analysis, to develop potential diagnostic tools.

Most of the FDA approved cancer biomarkers are single glycosylated proteins derived from serum (34). These include Cancer Antigen 125 (CA125) for ovarian cancer (17, 35), cancer antigen 19-9 (CA 19-9) for pancreatic cancer (36), cancer antigen 15-3 (CA 15-3) for breast cancer (37), prostate-specific antigen (PSA) for prostate cancer (15), and carcinoembryonic antigen (CEA) increases in colorectal, bladder, breast, pancreatic and lung cancer (38). These biomarkers are especially useful for monitoring disease in patients who have already been diagnosed, and in practice have had some value as diagnostics.

However, while many potential biomarkers are reported annually through mining of “omics” data, the transition from discovery to actual validation is relatively slow. Moreover, some of these approaches are useful in research but are expensive, require a specialized or centralized facility, and are difficult to translate commercially to provide a qualified large-scale service to populations. These are significant barriers for reliable patient access. Proteins and nucleic acids are not examined in this paper, as they have been the topic of many recent reviews.

## 3 Novel class of cancer biomarkers. Gangliosides

### 3.1 The nature of gangliosides

Gangliosides are a family of >40 different sialic acid-containing glycosphingolipids, with an overall organization of two lipid tails with a glycan tree (39). Ganglioside nomenclature is based on the specific glycan sequence and connectivity which is structurally unique, and defines each ganglioside by name in all species (40).

**Figure 1:**
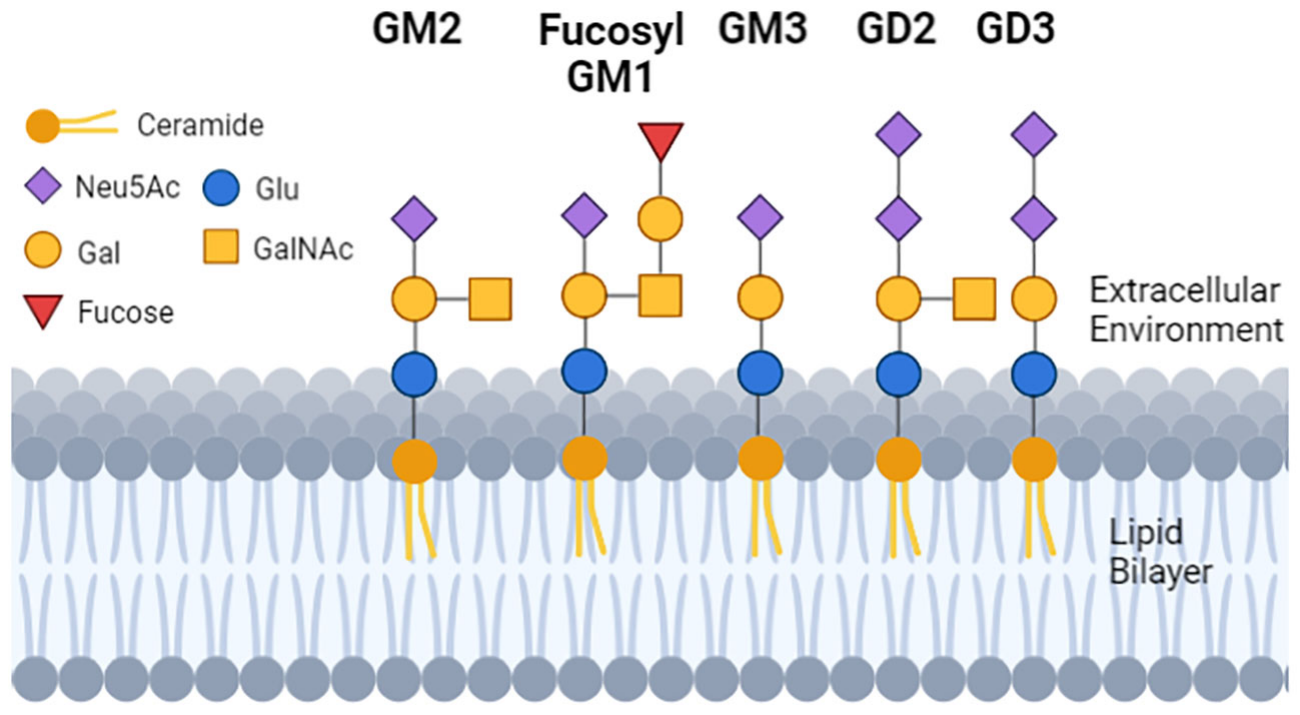
The structure and location of gangliosides in cells. Each geometric shape represents a specific type of sugar. For gangliosides that are on the cell surface, the glycan tree is exposed to the extracellular environment, and is linked to a ceramide (dark orange ball with two lipid tails embedded in the lipid bilayer). The sugar head of normal GM1 and the tumor GD3 differ by 2 sugars, and GD2 and GD3 differ from each other by 1 sugar. GM3 has a single N-Acetylneuraminic acid (mono sialylated) and can be a substrate to make GD3 (di-sialylated). GD3 can be a substrate to make GD2, so GD2 and GD3 can be (but not always) detected on the same cell.

The glycan moiety of the glycolipid is water soluble and extracellular (**Figure 1**). The sugar head is linked to a ceramide with two lipid tails embedded in the outer leaflet of membranes (41). In that location gangliosides regulate membrane fluidity (42), formation of membrane rafts and size (43–45) and within rafts promote the inclusion or exclusion of signaling proteins.

Within a ganglioside, the lipid tails are variable in their physical features such as carbon length and saturation state. The biological significance of the lipid tail heterogeneity is unknown (46). It is likely that the lipid tail variability has biological significance; and we proposed to exploit this heterogeneity diagnostically (47). For example, a cancer may be diagnosed based on a specific ganglioside carbohydrate head as a marker, but additional data mining can be obtained by evaluation of that ganglioside’s lipid tail length (or a range of lengths) (47).

Gangliosides such as GM1 and GM3 are ubiquitous in most cells including healthy cells. Other gangliosides such as GD2 and GD3 are defined as tumor marker gangliosides (TMGs), as they are low/absent in post-embryonic normal cells (46, 48) but are expressed at high levels by tumor cells (39, 49–53). TMGs are similar to carcinoembryonic markers, as they expressed at high levels during embryonic development, and shortly after birth TMGs are mainly absent in healthy cells (other than in a subset of normal adult neurons). TMGs are re-expressed in many types of cancers (39, 50, 51, 53) and cancer stem cells (54, 55) and appear to be implicated in cancer recurrence and cancer resistance to therapy; making them attractive therapeutic targets (56, 57).

### 3.2 The biosynthesis and location of gangliosides

**Figure 2:**
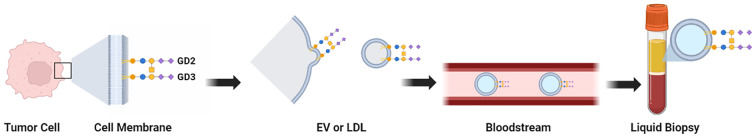
Graphical summary of the locations and physical forms of TMGs. Tissue biopsies can be evaluated by immunohistochemistry when specific mAbs against a TMG are available. Liquid biopsies can be evaluated by ELISA after extraction of gangliosides from the patient sample (when specific mAbs against a TMG are available), or by assays that quantify the presence of sialic acids. The full set of TMGs (hereafter, the Cancer Gangliosome) can be evaluated by LC-MS without mAbs. Purification of extracellular vesicles or exosomes from liquid biopsies may be an additional purification step to remove interference and to increase sensitivity as the TMGs are present in this compartment.

Synthesis of gangliosides begins in the endoplasmic reticulum and then continues in the Golgi apparatus, where sugars are added or removed by specific glycosyltransferases or glycanases (3, 52, 58). Gangliosides are transported to the outer leaflet of the cell membrane (46). TMGs in cancer cells have been reported to be shed (59) or secreted as micelles or in the form of extracellular vesicles, and may be incorporated within lipoprotein complexes (60–65). The TMGs can therefore be found at the tumor cell surface and also in three forms in bodily fluids: EVs, micelles, and lipoproteins (66). These released TMGs -in whatever form they are present in bodily fluids such as blood- may be potential high value biomarkers for diagnostics, as they are accessible in liquid biopsies (**Figure 2**).

### 3.3 Tumor marker gangliosides are present on extracellular vesicles

Cells release large volumes of vesicles from membranes or other organelles, including relatively large (30 to 150 nm) EVs that are shed via the budding of the plasma membrane. EVs are secreted into bodily fluids (e.g. CSF, blood, urine) and are relatively stable in those fluids until they are cleared by the liver or they become lodged in a specific organ. EVs represent an important mode of intercellular communication and a vehicle for transfer of membranes, proteins, mRNA and miRNA between cells (61). Cancer cells use EVs for immune modulation and to remodel the environment of a specific tissue to facilitate tumor cell metastasis (63, 67, 68) thus, EVs play an important role in oncogenesis (69–72).

TMGs are shed or are subject to vesicular release into circulation (43, 60, 64, 73–77). Since is documented that EVs and the cargo within EVs play an important role in oncogenesis (69–71, 78, 79) it is likely that TMGs present in EVs will likely have a role in oncogenesis as well, as discussed next.

### 3.4 Oncogenic-promoting biological roles of tumor marker gangliosides

Membrane-bound as well as soluble or shed TMGs play many biological functions in cell-cell recognition, cell adhesion, immune inhibition, and signal transduction (41–44, 46, 80). These features make TMGs etiological biomarkers because they are functionally implicated in cancer progression. TMGs provide tumors with advantages in growth/metastasis and immune evasion. The mechanisms described below link functional expression of TMGs with cancer progression.

First, TMGs on the cancer cell membrane activate signals promoting cellular growth [reviewed in (39, 51, 57)]. For example, expression of TMGs can lead to lowering of the threshold for activation of several wild type receptor tyrosine kinases (e.g. platelet-derived growth factor receptor, Trk receptor, and epidermal growth factor receptor) (81–91), and activation of soluble tyrosine kinases (such as p60^Src^, p56^Lck^) (81, 85, 92–95). Hence, oncogenic activation by TMGs is independent of growth factors.

This TMG-promoted dysregulated kinase activity is pro-oncogenic. Thus, TMGs may be etiological in tumors where mutations (or mutations of the activated kinases) are not clearly identified or are not a primary event. In addition, VEGF-mediated angiogenesis can be regulated *in vivo* by a change in TMG ratios. In this manner shed tumor gangliosides may promote tumor progression or facilitate metastasis by enhancing a leaky neo-vasculature and providing the necessary blood supply that enables tumors to extravasate and metastasize (96, 97). Pro-metastatic signals are further enhanced through modulation of adhesion proteins, regulation of membrane rafts, cell-cell and cell-matrix interactions (39, 57, 83, 85, 98–102).

Second, TMGs act functionally as Immune Checkpoint Inhibitors (ICI), to cause local and systemic immunosuppression, allowing the tumor to evade immune surveillance (103). Cancers inhibit or evade the immune system, by coopting a normal ICI process for immune regulation, examples comprising PD-1, CTLA4, and other proteins (104). Indeed, therapeutic strategies that target TMGs [such as anti-GD2 monoclonal antibodies (mAbs)] appear to reverse cancer resistance to therapies blocking an ICI protein PD-1 (1, 105). These data suggest that when a TMG is present, it may replace or potentiate the ICI function of PD-1 (52, 93, 106, 107). Circulating TMGs further suppress antigen presentation and immune activation systemically (i.e., throughout the body and not just in the local environment of the primary tumor) (108).

In sum, TMGs such as GD2 and GD3 suppress antigen presentation (87, 103, 109) and T cell immunity (108) locally and systemically, facilitating metastasis (68, 69, 71). This is advantageous to the tumor because systemic immunosuppression prevents secondary anti-tumor immune reactions against tumor neoantigens. Glycans containing sialic acids are a segment of the glycocode (110–112), and act as part of the glyco-immune-checkpoint, a new category of immune checkpoint blockade.

## 4 Expression of tumor marker gangliosides in cancer

**Table 1** presents publications that have reported expression of specific TMGs in certain cancers.

**Table 1:** Matrix of tumor associated gangliosides in cancer.

Cancer	GD2	GD3	Fucosyl GM1	GM2	GM3	PolySia	Siayl Lewis X	GT1b GD1a/b	GB3/CD77
Neuroblastoma	(41, 81, 113–117)	(114, 117)	(118, 119)	(114, 120, 121)	(122)	(114, 123, 124)			
Bladder					(86)				
Breast	(54, 125–127)	(125, 128, 129)	on EVs (130)	(114, 125)	(125)	(131)	(132, 133)		
Ovarian	(134)	(135)	(136)	(114, 136)	(137)				
Prostate				(114)					
Esophageal							(132)		
Head and Neck							(132, 133)		(74)
Non-small Cell Lung (NSCLC)			(138)	(114, 139)	(114, 140, 141)	(142, 143)	(114, 132, 133)		
Small Cell Lung (SCLC)	(87, 102)	(87, 102)	(56, 87, 102, 114, 138, 144)	(87, 102, 114)		(114)		(87, 102, 140)	
B cell Lymphoma	(114)			(114)					
Glioma	(145)	(90, 146, 147)		(148, 149)	(150–152)	(153)			
Pancreatic				(114, 154)		(114)	(132)		
Endometrial				(114)		(114, 148)			
Melanoma	(114, 117)	(114, 117, 155) (85, 99, 102, 105, 156, 157)	(114, 158)	(114, 159)	(48, 114)				
Soft Tissue Sarcomas	(160)	(160)		(114)					
Osteosarcoma	(83, 161–164)	(83, 162)		(114)					
Ewing’s sarcoma	(165, 166)	(162)		(114)	(167)				
Desmoplastic Round Cell	(168)	(162)		(114)					
Rhabdomyosarcoma	(162)	(162)		(114)					
Retinoblastoma	(169)				(170)				
Wilms tumor					(167, 171)	(172)			
Medullary Thyroid						(173)			
Stomach/Gastric				(114)		(114)	(132)		
Colon				(114)			(132, 133)		
Renal cell			(174)	(175)	(171)		(176)		

Summary modified and expanded from (50) and (112).

Tumor Marker Gangliosides have been listed among the most valuable cancer biomarkers (53). TMGs are expressed *homogeneously* at very high levels in both primary and metastatic nodules, decorate a wide variety of cancers, and are expressed uniformly across nodules within an individual patient (41, 177). Furthermore, in contrast to some other biomarkers, TMGs do not mutate or downregulate, even after chemotherapy (41, 113, 178–181).

## 5 Historical barriers limiting exploitation of tumor marker gangliosides for cancer diagnosis

To date, there are no approved or standardized diagnostic tests that exploit TMGs, nor has any been published techniques to measure TMGs quantitatively in liquid biopsies. The human cancer phenotypic studies presented in **Table 1** predominantly evaluated the expression of 1 or 2 TMGs per study using immunohistochemical (IHC) analysis of expression in tissue. Although IHC is a useful method for evaluating expression of novel markers, it is an inefficient and non-quantitative method. Therefore, there is a paucity of laboratory methods and very few specific antibodies fit for evaluating and quantifying TMGs, limiting their use to develop diagnostic methods (50). Thin Layer Chromatography (TLC) (85, 105, 156, 182) or lipid-associated sialic acids (LASAs) (183) have so far yielded only estimates of expression and often produced contradictory results. Even after 50 years of research worldwide, there are only a handful of monoclonal antibodies against TMGs (81, 146, 157, 184), and many are cross-reactive or poorly characterized (99, 102, 185). Since TMGs are glycolipids that can be generated via multiple biosynthetic pathways and enzymes (39, 51, 55, 147), monitoring mutations or mRNA levels does not appear to be feasible for robust diagnostics. These logistical challenges have limited the evaluation of TMGs for diagnostic purposes in cancer (4). It is noteworthy, however, that TMGs are validated targets for therapy and they represent an important source for developing cancer therapies [recently reviewed in (50, 186)].

### 5.1 Ovarian cancer

Ovarian cancer is the most fatal gynecological cancer, and lacks reliable diagnostics because there are no methods for early detection that could provide a clinical benefit (7). GD3 has been isolated from the polar lipid fraction of ovarian cancer-associated ascites, and identified to inhibit activation of natural killer (NK) cells. *In-vivo*, GD3 administration also dose-dependently inhibited natural killer T (NKT)-activation (135). These findings suggest that ovarian cancer tumors use GD3 to inhibit antitumor natural killer T-cell response as a potential mechanism for tumor immune evasion (80).

Recently, our group published an ELISA test that quantifies GD2 and GD3 in blood. We reported detection of 97% of early stage and late-stage ovarian cancer cases including those missed by the current standard clinical test CA125 (4). This was the first demonstration that TMGs can be accessed for quantitative diagnosis in a liquid biopsy. It was first used for ovarian cancer due to a significant unmet clinical need. Current work is expanding the test for diagnosis of other cancer such as melanoma and expanding the scope of the TMGs quantified to comprise the most relevant in **Table 1**.

### 5.2 Neuroblastoma

Neuroblastoma is the most common extracranial solid tumor in children. Neuroblastoma tumor tissues express GD2, which appears to be a marker of high-grade malignancy requiring more aggressive therapies. GD2 levels are significantly elevated in the sera of children with neuroblastoma compared healthy children and children with other cancers (59, 65). Furthermore, longitudinal evaluation of GD2 serum levels in patients showed a positive correlation with disease progression (113), indicating the potential for monitoring disease progression or the recurrence of neuroblastoma. However, GD2 levels were not quantified and further study is needed to determine the diagnostic validity. Evaluating combined expression of GD2 and GD3 in pediatric solid tumors, using radiolabeled monoclonal antibodies and tomography, has been suggested as a companion diagnostic to immunotherapy (162). One radiolabeled mAb is used for PET imaging to identify refractory neuroblastoma, but is not approved as standard of care (187).

### 5.3 Melanoma

GD3 is expressed on human melanoma cells and can modulate immune cell cytotoxicity (182) via siglec-7-dependent and -independent mechanisms (105, 156). Immunosuppressive function in melanoma was reported for GD2 and GD3 (80, 105, 156, 182). TMGs allow for more growth and motility and cellular invasion (99, 102, 157). GD3 is highly expressed in melanoma and regulates Src kinases and Yes kinases (81, 85, 157), likely through rafts, although specific mechanisms remain unidentified.

Given that the majority of human melanoma cases are positive for a TMG (mainly GD3 and GD2 have been evaluated) the possibility of using TMG markers for melanoma diagnosis or prognosis remains of high interest. Given the immunosuppressive role of some TMGs, which exhibit many of the features of immune checkpoint inhibition (ICI) such as PD1/PD1-L, it is also reasonable to hypothesize that TMGs may be at least partially involved in the known lack of response to ICI-blockade therapies in melanoma. This concept has been tested in neuroblastoma (188, 189). Evaluation in melanoma would provide with a diagnostic prediction of likely response to the standard of care ICI-blockade therapies, and this work is ongoing.

### 5.4 Bladder cancer

Bladder cancer is ranked as the fourth most common cancer in men, with occurrences four times greater than in women. High levels of GM3 have been observed to lower tumor cell motility and invasiveness and lower levels are able to increase cell motility and invasiveness (86). Therefore, in this circumstance, high levels of the specific GM3 ganglioside can be beneficial. Whether the presence of GM3 may related to lower presence of GD3 is an interesting hypothesis.

### 5.5 Bone cancer- osteosarcoma

GD2 is highly expressed in osteosarcoma tissues. *In vitro*, osteosarcoma cell lines generally expressed high levels of GD2 and GD3 (83, 164). GD3- and GD2-positive cells showed the most malignant properties. Therefore, GD2 may have a stronger phenotype than GD3 alone. Patient samples obtained during recurrence of cancer showed a higher intensity of GD2 staining compared to samples obtained during the initial biopsy. This indicates a notable increase in GD2 levels from the time of initial diagnosis to the recurrence of the disease (164).

### 5.6 Glioma and glioblastoma

A high percentage of glioma and glioblastoma tissues express GD2 and/or GD3. In gliomas, a large proportion express 9-O-acetyl GD3, or GD3 with an additional acetyl group on the terminal sialic acid, and the ratio of GD3 to 9-O-acetyl GD3 may be associated with enhanced tumor survival (147) or pro-tumorigenic events in glioblastoma (146).

### 5.7 Breast cancer

Several studies have suggested that GD2 and GD3 are involved in the development of various breast tumor types but the functional relationships between ganglioside expression and cancer development are not fully understood. GD3 is upregulated in approximately half of all invasive ductal breast carcinoma cases (127). One study showed that GD2 was highly expressed in a cohort of aggressive breast cancer subtypes, such as triple-negative and metaplastic (126), but confirmatory studies are needed. GD2 was also identified as a cancer stem cell-specific marker from human breast cancer cell lines and patient samples (54), and GD3 was associated with activated EGFR signaling in both breast CSCs and breast cancer cell lines (127).

GM3 was reported in the serum of breast cancer patients (190) and GD2/GD2 synthase are reported in breast cancer stem cells (54, 127, 191, 192) that are often responsible for recurrence of the disease. The value of TMGs for breast cancer diagnosis may be important, but it is undefined at this stage and requires further research.

### 5.8 Head and neck cancer

Head and neck squamous cell carcinoma (HNSCC) is the sixth leading cancer in the world (7). Expression of the glycolipid globotriaosylceramide 3 (GB3) was positively correlated with progression of head and neck cancer (HNC). During malignant transformation of HNC cells, changes in GB3 expression suggests that this marker could be used to identify HNSCC early on (74).

### 5.9 Lung cancer/small cell lung cancer

In the United States, lung cancer is the third most common cancer, with the annual number of new cases steady over the past 20 years. Expression of glycosyltransferase genes important for biosynthesis of gangliosides is elevated in lung cancer above normal bronchial cells (87). Both non-small cell lung cancer (NSCLC) and small cell lung cancer (SCLC) express mainly GM2 and GM1, whereas only SCLCs express b-series gangliosides GD2, GD1b, and GT1b as well as the expected upregulation of the GD3 synthase gene required for biosynthesis (87, 102), and express fucosylated GM1 (Fuc-GM1) (56). Expression of GD3 synthase or GD2 synthase in SCLCs increased growth rates and invasiveness, and anti-GD2 antibodies induced apoptosis, indicating that GD2 has a functional role in malignant growth.

### 5.10 Soft tissue sarcomas

GD2 and GD3 expression are present on human soft tissue sarcomas, as assessed in IHC performed on 56 tissue samples, in which 93% of tumors expressed GD2 while 88% expressed GD3 (160). Specifically, GD2 is widely expressed among neuroectodermal tumors as well as adult sarcomas using IHC (168). The intensity of expression and localization varied among sarcomas of different histologic types (160).

### 5.11 Other cancers

The ganglioside GM2 is highly expressed in pancreatic ductal adenocarcinoma (PDAC) and Cholangiocarcinoma (193). GM2-positive PDAC cells exhibited higher growth rates and invasion and were present in three-dimensional cultures, including cancer stem cell-like cells. GM2 expression was associated with aggressive PDAC characteristics, indicating its potential as a diagnostic target for PDAC. Cholangiocarcinoma, a bile duct epithelium malignancy prevalent in Southeast Asia and a significant health concern due to poor prognosis (194). This may be useful since early detection is challenging as the current serum marker (CA19-9) is insufficient due to low sensitivity/specificity (195).

## 6 Looking to the future of diagnosis

The data above provides evidence that GD2/GD3 and other TMGs may be present in different cancers at different ratios and with some degree of cancer specificity. Evaluating GD2/GD3 expression has been suggested as a companion diagnostic for utilizing immunotherapy targeting gangliosides for therapy (162). Other studies examined other solid tumors osteosarcoma, rhabdomyosarcoma, Ewing family of tumors, desmoplastic small round cell tumors, and melanoma (117, 160, 168, 184). Further, there were higher GD3 expression levels than GD2 among the other tumors analyzed. Recently, our group quantified GD2 and GD3 in blood as diagnostic of all stages and all forms of epithelial ovarian cancer with detection of 97% of early stage and late-stage ovarian cancer cases including the 40% of cases missed by the current test CA125 (4).

As mentioned above, TMGs are shed into the serum and thus may be utilized for diagnostics using liquid biopsies. However, it is noteworthy expression of one ganglioside does not dictate the fate of having a cancer, but a collection of tumor-gangliosides and their proportions or ratios may have important implications in terms of providing functional advantages for cancer growth or aggressiveness (50) and may be exploited for therapy or for diagnostics. Overall, the data suggests a strong rationale for utilizing a combination of TMGs as biomarkers for early stage detection of cancer and the varying ganglioside patterns/ratios among cancers and cancer types. This is discussed in the next section.

### 6.1 TMGs as pan-cancer diagnostic markers?

As indicated in **Table 1**, each tumor may express a limited number of TMGs, but TMGs are markers of a large number of tumor types. This intriguing finding prompted the question of whether it is possible to explore TMGs as a pan-cancer diagnostic. Pan-cancer detection may be a tool that can lead to early suspicion, and then to a genuine diagnosis using tests that detect one tumor-at-a-time. Cancer suspicion and eventual diagnosis often results from patients presenting to a physician with symptoms. When patients present with generic or confounding symptoms (like pelvic pain for ovarian and gastrointestinal cancers) a bona fide diagnosis is more difficult (26, 196). Moreover, there are asymptomatic individuals that do not visit a health care professional. We believe that an affordable pan-cancer diagnostic tool may be of high utility for these populations as well as potentially as a routine screening tool leading to earlier diagnosis.

Many companies are pursuing the goal of attempting to commercialize pan-cancer screens. Emerging tests have been reported to detect up to 3 or 4 tumor types based on nucleic acids mutations or methylation states. So far, tests fall below the expected threshold diagnostic coverage of over 50% of the patients in the top 30 tumor types. Moreover, the efficacy of these methods at detection of early stage cancers (when curative therapy is most likely) is low (26). Current studies utilizing circulating tumor DNA (ctDNA), and circulating miRNA are investigating the rigor and reproducibility of attempts to demonstrate high sensitivity and specificity from innovative diagnostic assays for early stage cancer detection, i.e. CancerSEEK (26). CancerSEEK detected cancer with a sensitivity of 69 to 98% (depending on cancer type) and 99% specificity from a study of 1000 patients previously diagnosed with cancer and 850 healthy control individuals as per their criteria. Although the specificity is high, the sensitivity has a 29% difference from its minimum to maximum percentage detected, suggesting a need for improvement prior to fully distributing and utilizing CancerSEEK among average- to high-risk populations. For circulating miRNA biomarker screening, studies are costly and reported studies are of small sample sizes leading to issues of reproducibility (197). Moreover, unfortunately, both ctDNA and miRNA are unstable and require specialized handling that is not feasible for many healthcare providers (198).

We note that there is still wide debate in the field as to the real clinical usefulness of a pan-cancer diagnostic tool; and the outcome measures, the analytical endpoints, and the preponderance of late stage cancer samples analyzed during the development of these techniques have been criticized (4, 199–202). That being said, identification of pan-cancer biomarkers that have potential to overcome the challenges will resolve an urgent and unmet need (203). This is an evolving field and implementation of pan-cancer tests or screening tools are still at a relatively early phase of development and it remains to be tested in the marketplace (e.g. can they be commercialized successfully over a long period of time).

Given that a large panel of TMGs are accessible in liquid biopsies, and they have ideal features for biomarkers, innovative methods to quantify all TMGs in liquid biopsies may facilitate a pan-cancer method or diagnostic tool. Cancer specificity may arise from the relative ratios of TMGs in a patient. We refer to this diagnostic ratio of TMGs as the Cancer Gangliosome, which may be a novel family for developing a pan-cancer diagnostic. In addition to TMG ratios, several features of the Cancer Gangliosome may be analyzed for developing diagnostic biomarkers. As stated above, the ganglioside lipids are variable in length, saturation, and oxidation states (15). The relevance of ganglioside lipid tail heterogeneity (or changes thereof in disease states) is of interest given that that differences or changes in lipid tail may have biological significance, for example affording different properties to signaling rafts or to the fate of exosomes. Hence, we proposed exploiting lipid heterogeneity diagnostically (47), as it is possible to exploit not only a specific carbohydrate as a diagnostic marker (e.g. GD2) but also to exploit the specific lipid tail length of that GD2 carbohydrate species to enable diagnostic specificity. As quantification of GD2 may be diagnostic of melanoma or glioblastoma or ovarian cancer (see **Table 1**), quantification of GD2 together with identification of a specific lipid tail length (or range thereof) or other physical features of the tail (such as oxidation or saturation state) can discriminate diagnostically between melanoma or glioblastoma or ovarian cancer.

## 7 Conclusions

TMG glycan trees provide for stable, invariant, and non-mutating cell surface markers, their expression is uniform in cancer nodules (41), does not downregulate in the surviving tumor cells after chemotherapy, and the biomarkers are functionally etiological to cancer (52, 163, 179, 204). The glycan markers are detectable in tissues (50, 205) and also in liquid biopsies which provides advantages for sampling (4). In addition, the TMG lipid tail lengths or other physical features may provide for additional parameters to develop diagnostic tools (47).

Early stage detection of cancers is essential to improving patient survival and quality of life. Exploiting a new family of tumor gangliosides is a promising addition to the menu of markers for diagnosing early stage cancers using a simple non-invasive blood test. In patients presenting to a healthcare provider with symptoms, an analysis of relevant TMGs can be used to aid diagnosis. Future work should be focused on the quantification of glycolipids and ganglioside presence (or relative ratios) in blood for potentially diagnosing patients at early and late-stages of cancer.

Here we propose that the Cancer Biomarker Glycocode family and the Cancer Gangliosome as a class which is poorly exploited in diagnosis. These markers are complementary to the more commonly exploited families of protein or nucleic acid markers, and can add to the repertoire of available markers, leading to innovation in cancer diagnosis and screening. Having available a diversity of biomarkers will help to alleviate the serious unmet clinical diagnostic needs, potentially accelerate treatments, lower morbidity, lower mortality, and reduced healthcare costs.

## Author contributions

AN: Conceptualization, Writing – original draft, Writing – review & editing. SY: Writing – original draft. AJ: Conceptualization, Writing – review & editing. HS: Conceptualization, Writing – review & editing, Funding acquisition, Writing – original draft.
